# Comprehensive Chemical and Sensory Assessment of Wines Made from White Grapes of *Vitis vinifera* Cultivars Albillo Dorado and Montonera del Casar: A Comparative Study with Airén

**DOI:** 10.3390/foods9091282

**Published:** 2020-09-12

**Authors:** José Pérez-Navarro, Pedro Miguel Izquierdo-Cañas, Adela Mena-Morales, Juan Luis Chacón-Vozmediano, Jesús Martínez-Gascueña, Esteban García-Romero, Isidro Hermosín-Gutiérrez, Sergio Gómez-Alonso

**Affiliations:** 1 Regional Institute for Applied Scientific Research (IRICA), University of Castilla-La Mancha, Av. Camilo José Cela, 10, 13071 Ciudad Real, Spain; jose.pnavarro@uclm.es; 2 Instituto Regional de Investigación y Desarrollo Agroalimentario y Forestal de Castilla-La Mancha (IRIAF), Ctra. Albacete s/n, 13700 Tomelloso, Spain; pmizquierdo@jccm.es (P.M.I.-C.); amenam@jccm.es (A.M.-M.); jlchacon@jccm.es (J.L.C.-V.); jmartinezg@jccm.es (J.M.-G.); estebang@jccm.es (E.G.-R.); 3 Parque Científico y Tecnológico de Castilla-La Mancha, Paseo de la Innovación 1, 02006 Albacete, Spain

**Keywords:** white wines, color, phenolic composition, volatile aroma compounds, odor activity values, sensory profile

## Abstract

The ability to obtain different wines with a singular organoleptic profile is one of the main factors for the wine industry’s growth, in order to appeal to a broad cross section of consumers. Due to this, white wines made from the novel grape genotypes Albillo Dorado and Montonera del Casar (*Vitis vinifera* L.) were studied and compared to the well-known Airén at two consecutive years. Wines were evaluated by physicochemical, spectrophotometric, high-performance liquid chromatography–diode array detection–mass spectrometry, gas chromatography–mass spectrometry and sensory analyses. The chromatic characteristics of the new wines were defined by more color purity than Airén, with greenish highlights. In general, the phenolic profile of the Albillo Dorado wines showed a higher flavonol and hydroxycinnamic acid derivative content. Several volatile compounds were determined, and their odor activity values were calculated to determine their impact on wine aroma. A fruity series dominated the wine aromatic composition, but spicier and greener notes characterized the aroma profile of Airén wines. Albillo Dorado and Montonera del Casar were sensory evaluated as wines with a less fresh taste compared to Airén. Unique chemical and sensory profiles were determined for wines made from these novel grape genotypes, providing alternative monovarietal wines to encourage the wine market growth and extend the offer to consumers.

## 1. Introduction

Dominated by the main international grape cultivars, the development of different white wine types is mostly determined by wine consumer preferences and needs in the global market [1]. White wines with specific organoleptic characteristics have been requested in the last years, being the most appreciated those that are characterized by varietal aromas, with green and tropical fruit notes and an elegant acid taste [2]. Wine sensory attributes are defined by their physical and chemical composition, mainly phenolic and volatile compounds, which play an important role in wine quality. White wines typically show a lower content of phenolic compounds than red ones [3]. Their polyphenolic fraction is usually dominated by hydroxycinnamic acid derivatives, hydroxybenzoic acids, flavonols and flavan-3-ols; it has been demonstrated that these compounds are related to wine sensorial properties, such as the chromatic characteristics, color stability, bitterness and astringency [4]. Regarding volatile compounds, terpenes and norisoprenoids are associated with grape cultivars and may be important for the expression of wine varietal characteristics [5].

Different styles of white wine elaboration have been developed in the last years, with the purpose to diversify the wine market. Some alternative practices to obtain wines characterized by singular sensorial properties are the fermentation and ageing in a barrique on lees [6], fermentation by endogenous yeast microflora [7] or prolonged maceration during and after fermentation and ageing in wooden barrels [8]. The use of indigenous or novel grape cultivars is another feasible option to make singular white wines. Recently, there is an increase of research works about indigenous grape cultivars from different countries such as Greece, Italy, Japan and Spain [9,10,11,12].

In south-central Spain, Castilla-La Mancha is the biggest wine area in the world, with 470,000 ha of vineyard. Conformed by several tens of indigenous grape cultivars and a significant number of foreign ones, the vine heritage of this region is going through a period of impoverishment due to the inclusion of foreign cultivars that may result in the autochthonous grape extinction. Because of the plant material loss, Instituto Regional de Investigación y Desarrollo Agroalimentario y Forestal (IRIAF) of Castilla-La Mancha carried out a vineyard prospective study to recover and characterize the indigenous grape cultivars of this region. That work allowed the identification of more than 40 new grape genotypes not previously described in the literature consulted [13,14]. The Grapevine Germplasm Bank of Castilla-La Mancha (GGBCM) was created to preserve the great diversity of grape varieties from this region, where they grow at optimal virus-free conditions. Albillo Dorado and Montonera del Casar are two novel white grape genotypes (*Vitis vinifera* L.) recently identified in Castilla-La Mancha, and they were found in different areas of this region: Albillo Dorado grapes are located in Albacete, and the Montonera del Casar genotype is distributed in several zones, i.e., Cuenca, Guadalajara and Toledo. Recently, the flavanol glycoside content of the grape seeds and skins of these novel genotypes was studied [15]. In addition, wines made from several minor grapes have been previously studied in Castilla-La Mancha, whose cultivation is restricted to small areas. For example, the volatile and sensory profile of wines elaborated with the Moravia Agria grape cultivar and the phenolic composition of Moravia Dulce, Rojal and Tortosí wines have been reported in the literature [16,17]. However, there is no known data about the physicochemical characteristics of wines made from these two novel grape genotypes.

The knowledge of the oenological potential of alternative grape cultivars can give opportunities to obtain distinctive wines that appeal to a broad cross section of consumers. Therefore, the aim of this work was to perform a detailed characterization of young white wines made from the novel grape genotypes Albillo Dorado and Montonera del Casar (*Vitis vinifera* L.) recently identified in Castilla-La Mancha, at two consecutive years (2015 and 2016). The study included the comparison to Airén, currently the most cultivated grape variety in this region and one of the most important white grapes in the world. Wines were assessed by physicochemical, spectrophotometric, high-performance liquid chromatography–diode array detection–electrospray ionization-mass spectrometry (HPLC-DAD-ESI-MS/MS), gas chromatography–mass spectrometry (GC-MS) and sensory analyses.

## 2. Materials and Methods

### 2.1. Chemicals

Analytical grade chemicals (>99%) and Milli-Q water (Merk-Millipore, Darmstadt, Germany) were used. The analysis of phenolic compounds was performed using solvents of LC-MS quality (Fisher Scientific, Madrid, Spain): acetonitrile (CH_3_CN), formic acid (HCOOH) and methanol (CH_3_OH). The following GC-MS grade solvents were used for the volatile compound analysis: *n*-pentane (CH_3_CH_2_CH_2_CH_2_CH_3_) and dichloromethane (CH_2_Cl_2_) that were purchased from Scharlab (Sentmenat, Spain), and Fisher Scientific (Madrid, Spain) provided the ethyl acetate (CH_3_COOCH_2_CH_3_) and CH_3_OH. Phenolic compound identification and quantitation were carried out using commercial standards from Phytolab (Vestenbergsgreuth, Germany): (-)-epigallocatechin, (-)-gallocatechin, quercetin 3-glucuronide, *trans*-caftaric acid and *trans*-piceid. Procyanidins B1, B2 and B4, quercetin 3-galactoside, quercetin 3-rutinoside, kaempferol 3-galactoside, kaempferol 3-glucuronide, kaempferol 3-rutinoside, the 3-glucoside derivatives of quercetin, kaempferol, isorhamnetin and their aglycones were supplied by Extrasynthese (Genay, France). Moreover, (-)-epicatechin, (-)-epicatechin 3-gallate, (+)-catechin and *trans*-resveratrol commercial standards were used from Sigma-Aldrich (Tres Cantos, Madrid, Spain). *Cis*-resveratrol and *cis*-piceid were obtained from their *trans* isomers subjected to UV-irradiation (366 nm light for 5 min in quartz vials) in a 25% CH_3_OH solution. For identifying and quantifying the volatile compounds, several chemical standards were purchased from Extrasynthese (Genay, France), Fluka (Buchs, Germany), Sigma-Aldrich (Steinheim, Germany) and Merck (Darmstard, Germany). The 4-nonanol and 4-methyl-2-pentanol compounds were obtained from Sigma-Aldrich (Steinheim, Germany), both used as internal standards.

### 2.2. Winemaking

Albillo Dorado, Montonera del Casar and Airén grapes were cultivated in the same vineyard located in the GGBCM under the warm climate of this Spanish region. Grapes were harvested in two consecutive years (2015 and 2016), at the optimal ripening stage for winemaking: sugar content, 19.3–22.5° Brix; total acidity, 2.7–5.6 g L^−1^; and pH, 3.5–3.9. The winemaking was made in duplicate, employing 75 kg of each grape genotype to elaborate monovarietal white wines at the experimental winery of IRIAF. When grapes were destemmed and crushed, the addition of SO_2_ (80 mg L^−1^) protected the grape must from undesirable microorganisms. A cold pre-fermentative maceration was carried out at 5 °C during 24 h. The alcoholic fermentation started in stainless steel tanks (100 L capacity) by inoculation with *Saccharomyces cerevisiae* yeast (Uvaferm VN^®^, Lallemand Inc., Zug, Switzerland) at 20 g hL^−1^. The fermentation was conducted at 17 °C, controlled by density measure and finished when the glucose + fructose level was <5 g L^−1^. Afterwards, the wines were racked and adjusted to 25 mg L^−1^ SO_2_. The Battonnage technique was performed for one month, which consists of stirring the dregs regularly. Finally, the wines were stabilized at −5 °C for 15 days, corrected up to 25 mg L^−1^ SO_2_, passed through 0.2 µm filters, bottled and stored at 16–18 °C. The wines were elaborated and analyzed in their vintage years.

### 2.3. Wine Physicochemical and Spectrophotometric Analyses

The analytical methods proposed by the International Organisation of Vine and Wine (OIV) [18] were used to determine the following wine physicochemical parameters: alcoholic strength, relative density, total and volatile acidity, pH, glucose + fructose concentration, glycerin content, SO_2_ and the concentration of different acids. White wines were subjected to CIELab color space through the MSCV^®^ software [19] in order to obtain their chromatic characteristics. Total phenolic content was analyzed according to the method proposed by the Folin–Ciocalteu method [20] and a precipitation assay with methyl-cellulose was followed for the condensed tannin measurements [21].

### 2.4. HPLC-DAD-ESI-MS/MS Analysis of Flavonols and Hydroxycinnamic Acid Derivatives

The flavonols and hydroxycinnamic acid derivatives (HCADs) were analyzed on an Agilent 1100 Series HPLC system (Agilent, Waldbronn, Germany), equipped with a diode array detector (model G1315B) and mass spectrometry detector with an electrospray ionization source (LC/MSD Trap VL, model G2445C VL). Before analysis, 2 mL of the wine were dried in a rotary evaporator at 35 °C and reconstituted in 1 mL of 20% CH_3_OH. The sample was injected (40 μL) on a reversed-phase column ZORBAX Eclipse XDB-C18 (2.1 × 150 mm; 3.5 µm particle, Agilent USA, Santa Clara-California) at 40 °C. The chromatographic conditions used were as follows: Solvent A (CH_3_CN/H_2_O/HCOOH, 3:88.5:8.5 *v*/*v*/*v*), Solvent B (CH_3_CN/H_2_O/HCOOH, 50:41.5:8.5, *v*/*v*/*v*) and Solvent C (CH_3_OH/H_2_O/HCOOH, 90:1.5:8.5, *v*/*v*/*v*), with a flow rate of 0.19 mL min^−1^. For the HPLC analysis of these phenolic compounds, the solvent gradient was (time, % each solvent) zero min, 96% A and 4% B; 8 min, 96% A and 4% B; 37 min, 70% A, 17% B and 13% C; 51 min, 50% A, 30% B and 20% C; 51.5 min, 30% A, 40% B and 30% C; 56 min, 50% B and 50% C; 57 min, 50% B and 50% C; and 64 min, 96% A and 4% B. For identification purposes, the mass spectrometer was operated under the following conditions: negative ionization mode; scan range, 100–1000 *m*/*z*; dry gas, N_2_, flow 8 L min^−1^; drying temperature, 350 °C; nebulizer, 40 psi; capillary, +3500 V; capillary exit offset, −68 V; skimmer 1, −20 V; skimmer 2, −60 V. The identification of these compounds was based on their UV-Vis and MS/MS spectra, obtained from standards and matches such as those reported in the literature [22,23]. Flavonol quantitation was performed using DAD-chromatograms extracted at 360 nm. The total concentration of flavonols was expressed as equivalents of quercetin 3-glucoside. For the hydroxycinnamic acid derivatives, DAD-chromatograms at 320 nm were used and their total concentration was expressed as *trans*-caftaric acid equivalents (Table S1).

### 2.5. HPLC-MS/MS-MRM Analysis of Flavan-3-ols and Stilbenes

Flavan-3-ol and the stilbene fractions were obtained by solid phase extraction (SPE) using C18 cartridges (Sep-pak Plus C18, 820 mg of adsorbent, Waters Corporation, Milford, USA). The extraction and analysis of these compounds were carried out following the procedure described in the literature [24]. Separation, identification and quantitation of wine flavan-3-ols and stilbenes were performed on an HPLC Agilent 1200 series system (Agilent, Waldbronn, Germany) coupled to a DAD (Agilent, Waldbronn, Germany) and AB Sciex 3200 QTRAP triple quadrupole mass spectrometer with turbo spray ionization (Applied Biosystems, Foster City, CA, USA) (ESI–MS/MS) in the Multiple Reaction Monitoring (MRM) mode. Analyst MSD software (Applied Biosystems, version 1.5) was used to process the mass spectral data. The total flavan-3-ol monomer concentration was expressed as (+)-catechin equivalents, flavan-3-ol dimers were quantified as equivalents of procyanidin B1 and stilbene concentration was expressed as *trans*-resveratrol equivalents.

### 2.6. GC-MS Analysis of Volatile Aroma Compounds

Volatile compound extraction was obtained following the methodology reported in [25], using SPE cartridges (LiChrolut EN, Merck, 0.3 g of phase, Darmstadt, Germany) and an internal standard (4-nonanol, 0.1 g L^−1^). A volume of 25 mL of wine, containing the internal standard, were passed through preconditioned cartridges and then the resin was washed with 25 mL of Milli-Q water in order to remove the sugars and polar compounds. The volatile fraction elution was carried out with 15 mL of pentane-dichloromethane (2:1, *v*/*v*). Then, the extracts were concentrated to 150 μL by distillation in a Vigreux column and then under a nitrogen stream and kept at −20 °C until GC-MS analysis. Wine volatile compounds were determined using a gas chromatograph FocusGC coupled to a mass spectrometer ISQ with electron impact ionization source and quadrupole analyzer, and equipped with an autosampler TriPlus (ThermoQuest, Waltham, MA, USA). A capillary column BP21 (SGE, Ringwood, Australia) (50 m × 0.32 mm internal diameter; 0.25 µm film thickness; FFPA stationary phase, polyethylene glycol treated with nitroterephthalic acid) was used. For the analysis of major volatile compounds, 100 µL of wine was diluted with 100 μL of 4-methyl-2-pentanol (50 mg L^−1^) as the internal standard and 1 mL of Milli-Q water [26]. The sample was injected (0.8 µL) in split mode (split ratio: 10) at 195 °C. Helium was used as the carrier gas with a constant flow of 1.2 mL min^−1^. The oven temperature was 32 °C during 2 min, 5 °C min^−1^ to 120 °C, 75 °C min^−1^ to 190 °C and 18 min at 190 °C. Regarding the minor volatile compounds, 1 µL of the SPE eluate was injected in the splitless mode (splitless time: 0.3 min). The chromatographic conditions were as follows: oven temperature, 40 °C for 15 min, 2 °C min^−1^ to 100 °C, 1 °C min^−1^ to 150 °C, 4 °C min^−1^ to 210 °C and 55 min at 210 °C; injector temperature, 220 °C; and carrier helium gas, 1 mL min^−1^. The following detector parameters were set: mass scanning range, 40–250 amu; ion source temperature 250 °C; impact energy, 70 eV; and electron multiplier voltage, 1603 V. The mass-spectral library, retention times and pure commercial volatile compounds allowed the volatile compound identification. The specific *m*/*z* fragment for each compound was used for the quantification by GC-MS, using the internal standard method. The concentration of non-available commercial compounds was expressed as equivalents of the internal standard obtained by normalizing the peak area of each compound to that of the internal standard and multiplying by the internal standard concentration [27].

### 2.7. Wine Sensory Analysis

White wines made from the novel grape genotypes and Airén were sensory evaluated by 11 panelists with considerable experience in tasting wines (staff members from IRIAF trained in descriptive sensory analysis of wines, age range from 25 to 65, 60% female and 40% male), under ISO standards related to methodology and vocabulary, tasting room and taster selection and training [28,29,30]. The Napping^®^ technique was performed to determinate the organoleptic properties of wines [31]. According to similarities or differences in the sensory profile, the wine samples were placed on a white paper sheet of 40 cm × 60 cm without imposing structure or attributes, which allowed to identify the most important attributes for tasters. The results obtained from each wine sample were two coordinates (X and Y) that can be converted into a distance matrix. A further session was planned with a list of wine attributes imposed by the chief judge and previously chosen by an expert panel. The distribution of the wine samples on the blank sheet was established according to similarities and dissimilarities between wines with the attributes defined. Once the evaluation sessions finished, data obtained from each taster and wine sample provided the coordinates and frequency tables.

### 2.8. Statistical Analysis

All chemical data were treated using a one-way analysis of variance (ANOVA, Student–Newman–Keuls test, *p* < 0.05) to identify significant differences between the wines between the two studied years. Statistical tests were performed using SPSS software, version 23.0 (IBM, Endicott, New York, USA). Sensorial data were processed with XLSTAT 2017 statistical software (Addinsoft, Paris, France).

## 3. Results and Discussion

### 3.1. Physicochemical Parameters and Chromatic Characteristics

Table S2 shows the physiochemical data of white wines made from Airén and the novel grape genotypes. Alcoholic fermentation finished correctly as indicated by the low content of fructose and glucose (≤0.35 g L^−1^), being considered as dry white wines (reducing sugars < 5 g L^−1^). The optimal total acidity (3.7–5.8 g L^−1^), pH (3.4–3.6) and volatile acidity (0.2–0.4 g L^−1^) values were determined for all wines, with a suitable alcoholic content and within the usual parameters shown by white wines made in this Spanish region [32].

Regarding the chromatic characteristics, no significant differences were found between all evaluated wines for L*, which corresponded to lightness. The Montonera del Casar and Albillo Dorado wines provided greater color purity than the Airén ones (chroma, C*). Moreover, the parameter a* and b* measured for the new white wines differed from that observed in Airén, with higher b* values and more negatives for a*. This was manifested by a more yellow color with greenish highlights, an appealing feature for this type of wines.

The use of novel grape genotypes did not affect the total polyphenol content of the wines since no statistical differences were measured, accounting 300–410 mg L^−1^ as gallic acid equivalents. The new wines were characterized by a significant lower condensed tannin concentration than the Airén ones. Those data do not offer much information about the phenolic compound influence on the interesting sensory properties of the wines. Due to this, the detailed composition of several phenolic compounds classes was studied.

### 3.2. Phenolic Composition

The flavonol profile of the wines assessed is shown in Figure S1. The 3-glycoside series of quercetin aglycone were determined for all wines, comprising 3-glucoside, 3-galactoside and 3-glucuronide. In addition, the 3-glucoside and 3-galactoside derivatives of kaempferol and isorhamnetin were also identified, and the free aglycones released from them by hydrolysis in wine. Results obtained were in accordance with reported data for white grapes and wines, with the presence of the flavonols mono- and di-substituted in the B-ring [33]. The wine analysis showed the absence of the B-ring tri-substituted flavonols, which are myricetin, laricitrin and syringetin. As expected in white wines, the profile of the wines made from Airén and the novel grape genotypes was dominated by quercetin-type flavonols, with a proportion of 80–100% (Table 1). Kaempferol-based flavonols were the second more important ones found in the new wines (7–16%) and absent in the Airén ones. The Albillo Dorado wines were characterized by the exclusive presence of kaempferol- and isorhamnetin 3-galactosides.

Moreover, the flavonol fraction of the Airén and Albillo Dorado wines showed a compound with a molecular ion at *m*/*z* 447 in the MS spectrum that suffered the 146 amu loss in the ion trap, which results in a product ion at *m*/*z* 301, corresponding to the deprotonated moiety of quercetin aglycone. This allowed the identification of the compound quercetin 3-rhamnoside, previously reported in wines made from Moribel grapes [23]. A high degree of flavonol hydrolysis was determined in the wines, reaching 80% in Montonera del Casar. However, the flavonols were not hydrolyzed in the 2015 Airén wines, which can be explained by the low content of these compounds. The most abundant aglycone was quercetin, which is more polar and soluble in a hydro-alcoholic solution like wine. The total content of flavonols measured by HPLC accounted 0.1–11.4 mg L^−1^ as quercetin 3-glucoside equivalents. These values were low due to the winemaking process, since flavonols are mainly located in grape skins and their concentration in wines is dependent on skin extraction. Besides the abovementioned, the flavonol concentration of the studied wines was lower compared to other grape variety wines, such as Macabeo white wines from this region [34]. Although wine flavonols could be affected by many factors [35,36], grape variety also introduced variability in the total concentration of these compounds since all the studied wines were elaborated using the same conditions and winemaking techniques, and Albillo Dorado provided the flavonol-richest wines both years.

The monomeric flavan-3-ols determined in the wines are shown in Table S3. The (+)-catechin compound accounted for approximately half of the wine monomers, followed by (-)-epicatechin and (-)-gallocatechin. In addition, the glycosylated derivatives of the flavan-3-ol monomers were quantified in all the wines. Montonera del Casar wines were characterized by the lowest monomeric flavan-3-ol concentration (ca. 1.7 mg L^−1^ as (+)-catechin equivalents) and Albillo Dorado with values close to Airén (16 mg L^−1^) in 2016. The dimeric flavan-3-ol profile of the wines was dominated by procyanidin B1, with a higher proportion in the 2016 Montonera del Casar wines. A greater proportion of procyanidin B4 was found in the Airén wines and the galloylated dimers in the Albillo Dorado ones. Wines made from Airén grapes were characterized by the highest concentration of flavan-3-ol dimers, similar to monomers. These compounds and their polymers contribute to a mouthfeel sensation of white wines, such as bitterness and astringency [37].

Regarding the non-flavonoid compounds, hydroxycinnamic acids exist in wine as free form or esterified with tartaric acid. The expected hydroxycinnamoyl-tartaric acids (caftaric, coutaric and fertaric acids) were the hydroxycinnamic acid derivatives (HCADs) found in all wines, and also the ethyl ester of caffeic acid. Figure S2 shows the profile of these compounds in wines made from Airén and the novel grape genotypes. Corresponding to fertaric acid, the extracted ion chromatogram *m*/*z* 325 was also detected with different retention time and fragmentation pattern. This compound was a glucoside derivative of p-coumaric acid, previously reported in wines made from BRS Violeta grapes [22]. The identification of 2-S-glutathionylcaftaric acid (grape reaction product or GRP) was confirmed by UV-vis and MS/MS spectra. This compound is a reaction product of glutathione with oxidized caftaric acid, and its *trans* and *cis* isomers were found in all the wines. The formation of GRP avoids the browning of wines and also provides information of the oxidation suffered by the wine during its elaboration and aging [38]. With regards to the HCAD-types, the analyzed wines had greater caffeic-type proportions (39–60%), mainly *trans*-caftaric acid, except in the case of Montonera del Casar wines (Table 1). Statistical differences were observed in the total content of hydroxycinnamic acid derivatives among the wines. The highest HCAD concentration was determined in the Albillo Dorado wines—in both years. Data obtained in 2015 were in agreement with those reported for Chardonnay wines from Castilla-La Mancha [39]. 

Table S3 shows the stilbenes based on resveratrol identified in the studied wines. The main stilbenes were *cis*-resveratrol (32–62%) and *cis*-piceid, its 3-glucoside derivative (29–45%). Montonera del Casar provided wines with the lowest total stilbene concentrations, accounting around 0.08 mg L^−1^ as equivalents of *trans*-resveratrol and similar to those reported for *Vitis vinifera* white wines [40]. Nevertheless, these values were approximately half the concentrations in wines made from Albillo Dorado and Airén grapes (0.13–0.32 mg L^−1^).

### 3.3. Volatile Composition and Aromatic Series

Volatile compounds are crucial to the quality of wines due to their influence on the wine aroma profile. Concentrations of the most relevant volatile compounds on white wine aroma, their odor descriptors, aromatic series and thresholds are shown in Table 2. These volatile compounds belonged to different chemical families, e.g., acids, alcohols, aldehydes, benzenic compounds, C6 compounds, esters, furanic compounds, lactones, norisoprenoids and terpenes. Some of them have originated from grapes (varietal aromas) or were produced during alcoholic fermentation (fermentative aromas).

C6 compounds form part of the wine varietal aroma and are related to green and herbaceous nuances. They can provide undesirable flavors at high concentrations, having a negative impact on wine quality [41]. These compounds are considered one of the most important groups of varietal aromas in the wines assessed due to their concentrations. The main six carbon alcohol was *cis*-3-hexenol in wines made from Airén and Albillo Dorado grapes, accounting for 0.6–1.9 mg L^−1^. A lower concentration of its *trans* isomer was determined, in accordance with reported data for white wines [34]. However, the ratio between *trans*- and *cis*-3-hexenol moved in the opposite direction to Montonera del Casar. Wines were characterized by a higher total C6 compound content than other white wines elaborated with grapes grown in this region [39], and Airén exhibited the richest profile of these compounds. Other typical varietal aroma compounds are terpenes that provide citric and floral aromas [42]. Citronellol, geraniol and linalool were the main terpenes determined in all the wines. With values ranging between 2.9 and 6.8 µg L^−1^, the concentration of geraniol was generally the biggest and close to data reported for Chardonnay wines from Castilla-La Mancha [43]. In this work, terpene concentrations were under their threshold values, so the contribution of these compounds to wine aroma did not seem significant. Norisoprenoids are volatile compounds related to the varietal typicity of wines with low odor thresholds [42,44]. *β*-damascenone provided fruity aromas since its concentration exceeded the odor threshold value (0.05 µg L^−1^) [42] in all wines. Although there were no significant differences in total concentration of benzenic compounds between wines, several individual compounds differed between each other. The benzenic compound profile of Montonera del Casar wines showed higher guaiacol concentration in 2016 (11.8 µg L^−1^), with a value above its odor threshold (10 µg L^−1^) [45]. Moreover, it was characterized by having the greatest concentration of benzaldehyde in both years. The content of this compound has not exceeded its olfactory threshold (2000 µg L^−1^) [46] but it could have a synergic effect to wine aroma, contributing to fruity and floral notes. Related to sweet spice aroma, eugenol was found at a higher concentration in Airén wines. The content of this compound and vanillin did not exceed their odor threshold values [47].

During alcoholic fermentation, alcohols are formed as a byproduct of yeast amino acid metabolism. In isolation, these compounds do not typically have desirable odors and could be a negative factor on wine quality if their concentration is above 400 mg L^−1^ [48]. Since the total concentration of these compounds was under this value (284–345 mg L^−1^), they can make a positive contribution to wine complexity. Due to their higher solubility and volatility, mid-change fatty acids can have an important impact on wine flavor, providing fruity, cheese, fatty, and rancid notes. The studied wines had acid contents within the range 14–76 mg L^−1^, showing higher values in 2016 and with similar 6, 8 and 10-carbon fatty acid concentrations to those reported for Muscat wines [49]. The main esters found in wines were the ethyl esters and acetates of fatty acids. These compounds are key contributors to the fruity aromas of wines [50] and produced by yeasts in an enzymatic process during alcoholic fermentation. The new wines were characterized by higher ester concentrations than the Airén ones in 2015. Acetaldehyde is a wine aldehyde associated with fruity and nutty aromas and formed mainly by yeast metabolism during alcoholic fermentation, before ethanol formation. The wine acetaldehyde concentration depends on the yeast strain and initial must SO_2_ concentration; consequently, the higher acetaldehyde content in Airén wines can be due to the must composition since all the wines were made under the same conditions.

Among the main lactones determined in these white wines, *γ*-butyrolactone showed the highest concentrations with values below its odor threshold (35 mg L^−1^) [53]. Other lactones identified, such as *γ*-nonalactone and *δ*-dodecalactone, are also found in beer so they may be fermentation products.

**Figure 1:**
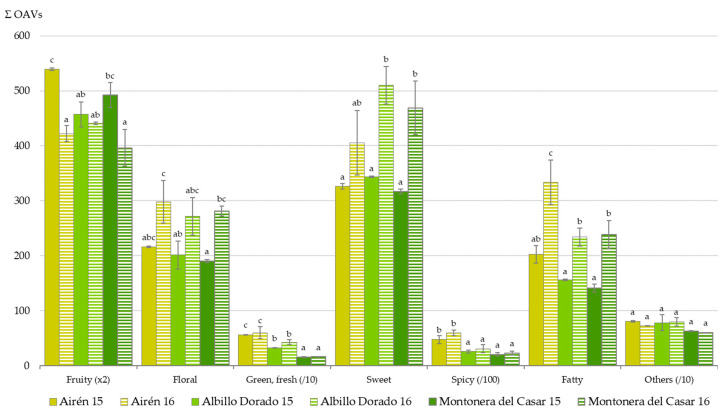
Aromatic series (ΣOAVs, mean value ± standard deviation, *n* = 2) in white wines made from Airén and the novel grape genotypes at two consecutive years (2015 and 2016). Different letters in the same aromatic series indicate that the values are significantly different among the wines (ANOVA, Student–Newman–Keuls test, *p* < 0.05).

Each volatile compound identified has a different impact on the overall aroma character of wines. To assess the influence of the aroma compounds in the studied wines, the Odor Activity Values (OAVs) were determined by dividing the compound concentration in the wine by the concentration corresponding to its odor threshold from the literature [42,45,46,47,51,52,53]. To estimate the overall wine aroma, the following aromatic series was established to bring together the volatile compounds according to their odor descriptors: green/fresh, fatty, floral, fruity, spicy, sweet and other odors. The values of the aromatic series were calculated based on summation of the OAVs for volatile compounds assigned to each series. The total intensity of each aromatic series (ΣOAVs) used to define the aroma profile of the white wines studied is shown in Figure 1. The highest aroma contribution to the wines was the fruity series that was the most characteristic attribute of the wine aromatic profile, followed by the sweet series. There are statistically significant differences in the intensity values of the green/fresh and spicy series between the wines, Airén showing the highest ones in both years. This could be explained by its C6 compound and eugenol concentrations, related to higher intensities of fresh and spice notes, respectively. The floral and green/fresh series were used by winetasters to define the sensory aroma profile of these wines although they are not the most abundant aromatic series. This issue can be attributed to the fact that the intensity of the sensory attributes of the wine aroma is affected by suppression, synergy and effects on the wine matrix, which is not taking into account in the OAV determination. 

### 3.4. Sensory Profile

**Figure 2:**
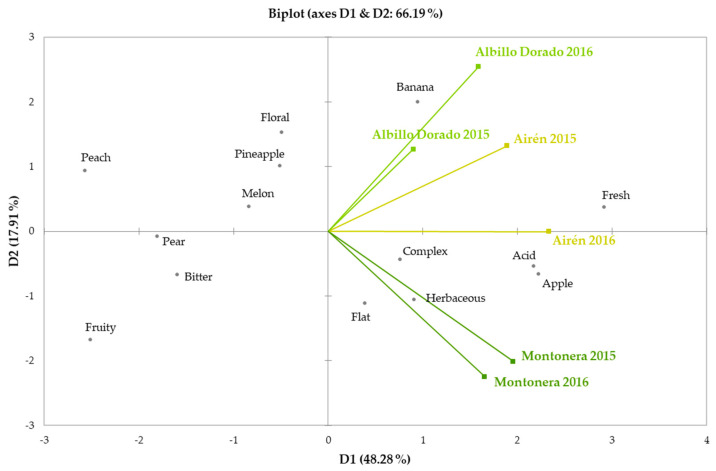
Sensory characterization of the white wines made from Airén and the novel grape genotypes at two consecutive years (2015 and 2016).

The sensory characterization of the wines made from Airén and the novel grape genotypes was performed using the Napping^®^ technique by experienced winetasters, professionals with great knowledge of wines. A simplified view of the sensory characteristics of the wines on a two-dimensional map is shown in Figure 2. The first two dimensions accounted for 66.19% of the explained variance (48.28% and 17.91%, respectively). According to the results, it was observed that each monovarietal wine produced from the two years, 2015 and 2016, appear close together, which indicates only little sensorial differences among the vintages. In addition, all wines were placed on the right side of the graph, showing that the wines were described by a somewhat similar sensory profile. However, each wine was defined by singular organoleptic properties. Airén wines were characterized by green apple notes, a distinguishing attribute in wines made from this grape variety [43]. Floral aromas with banana notes defined the sensory profile of the Albillo Dorado wines, similar to its homonym, the Albillo grape cultivar [49]. A herbaceous odor descriptor was correlated with the profile of Montonera del Casar wines. Concerning mouthfeel properties, wines made from the novel grape genotypes presented a less fresh taste and acidity than the Airén ones. The sensory assessment underlined the differences among the wines according to grape genotype, impacting on the global organoleptic quality in accordance with the chemical composition.

## 4. Conclusions

An exhaustive characterization of young white wines made from the novel grape genotypes Albillo Dorado and Montonera del Casar (*Vitis vinifera* L.) was performed in two consecutive years, comparing them to wines made from the Airén cultivar. Several significant differences in terms of chromatic characteristics and chemical composition (phenolic and volatile compounds) were established for the wines assessed. In addition, each wine was sensory characterized by singular organoleptic properties, with the positive and desirable attributes for these types of wines.

The data presented herein provide valuable information about the oenological potential of these novel grape genotypes for the first time. Moreover, the findings could be useful for the winemaking sector, mainly wineries and producers, in order to obtain distinctive monovarietal wines from these grape genotypes, increasing the diversification of white wines in the market and extending the offer to a broad cross-section of consumers.

## Figures and Tables

**Table 1:** Chromatographic and spectroscopic characteristics, molar proportions and total concentration (mean value ± standard deviation, *n* = 2) of the flavonols and hydroxycinnamic acid derivatives identified in white wines made from Airén and the novel grape genotypes.

				Airén		Albillo Dorado		Montonera del Casar	
Peak ^1^	RT (min)	Compounds	Molecular and Product Ions (*m*/*z*)	2015	2016	2015	2016	2015	2016
**Flavonols (mg L^−1^) ^2^**				0.14 ± 0.01 a	1.70 ± 0.74 a	4.53 ± 0.34 b	11.40 ± 1.60 c	1.93 ± 0.45 a	0.55 ± 0.02 a
1	26.21	Quercetin 3-galactoside	463, 301	ND	9.89 ± 2.73 b	7.08 ± 2.04 ab	5.55 ± 0.37 ab	4.60 ± 0.97 a	ND
2	26.74	Quercetin 3-glucuronide	477, 301	38.49 ± 9.56 b	ND	ND	ND	4.50 ± 2.14 a	4.64 ± 0.64 a
3	28.23	Quercetin 3-glucoside	463, 301	15.09 ± 1.48 ab	11.98 ± 3.74 ab	22.05 ± 0.19 c	17.41 ± 1.18 b	9.41 ± 0.82 a	12.57 ± 0.25 ab
4	31.95	Kaempferol 3-galactoside	447, 285	ND	ND	2.45 ± 0.46 b	1.57 ± 0.00 a	ND	ND
5	32.66	Quercetin 3-rhamnoside	447, 301	46.42 ± 8.08 b	3.72 ± 1.40 a	0.68 ± 0.05 a	0.60 ± 0.02 a	ND	ND
6	34.98	Kaempferol 3-glucoside	447, 285	ND	ND	2.56 ± 0.60 a	2.64 ± 0.19 a	ND	ND
7	36.65	Isorhamnetin 3-galactoside	477, 315	ND	ND	0.50 ± 0.17 a	0.37 ± 0.05 a	ND	ND
8	38.24	Isorhamnetin 3-glucoside	477, 315	ND	1.47 ± 0.12 a	2.29 ± 1.17 b	1.51 ± 0.11 a	1.26 ± 0.35 a	ND
9	42.98	Quercetin	301	ND	72.94 ± 7.74 b	52.02 ± 1.48 a	56.93 ± 1.96 a	72.98 ± 4.49 b	82.79 ± 0.89 b
10	51.68	Kaempferol	285	ND	ND	8.40 ± 2.48 a	11.34 ± 0.14 b	7.26 ± 0.15 a	ND
11	55.31	Isorhamnetin	315	ND	ND	1.96 ± 0.96 a	2.08 ± 0.70 a	ND	ND
		% Quercetin-type		100.00 ± 0.00 e	98.53 ± 0.12 d	81.83 ± 0.33 b	80.49 ± 0.43 a	91.48 ± 0.20 c	100.00 ± 0.00 e
		% Kaempferol-type		ND	ND	13.42 ± 2.63 b	15.56 ± 0.33 b	7.26 ± 0.15 a	ND
		% Isorhamnetin-type		ND	1.47 ± 0.12 a	4.76 ± 2.30 c	3.96 ± 0.76 b	1.26 ± 0.35 a	ND
		% Hydrolysis		00.00 ± 0.00 a	72.94 ± 7.74 bc	62.38 ± 3.00 b	70.36 ± 1.39 bc	80.24 ± 4.64 c	82.79 ± 0.89 c
**Hydroxycinnamic Acid Derivatives (mg L^−1^) ^3^**				50.53 ± 2.47 b	42.04 ± 0.97 a	66.05 ± 0.55 c	54.06 ± 1.42 b	41.45 ± 2.04 a	40.58 ± 1.96 a
12	2.15	Caffeoyl-glucose	341, 179, 161	11.11 ± 1.84 a	13.82 ± 1.23 ab	16.56 ± 3.08 bc	19.07 ± 0.49 c	30.15 ± 0.83 d	32.13 ± 0.68 e
13	3.46	*trans*-2-S-Glutathionyl-caftaric acid	616, 484, 440, 272	13.29 ± 0.45 a	13.43 ± 0.74 a	15.01 ± 3.89 a	17.62 ± 0.78 a	19.97 ± 1.13 a	46.19 ± 1.79 b
14	3.84	*trans*-Caftaric acid	311, 179, 149	42.22 ± 0.25 cd	37.48 ± 0.06 c	42.85 ± 4.87 d	32.60 ± 0.29 bc	29.20 ± 2.87 b	6.71 ± 0.43 a
15	4.43	*cis*-2-S-Glutathionyl-caftaric acid	616, 484, 440, 272	0.44 ± 0.07 a	1.27 ± 0.02 b	0.90 ± 0.18 ab	1.01 ± 0.21 b	0.87 ± 0.22 ab	1.25 ± 0.15 b
16	5.58	*trans*-Coutaric acid	295, 163, 149	11.62 ± 0.10 b	12.28 ± 0.27 b	9.86 ± 2.55 b	12.37 ± 0.55 b	3.69 ± 0.24 a	2.14 ± 0.31 a
17	5.74	*cis*-Coutaric acid	295, 163, 149	6.04 ± 0.39 c	5.12 ± 0.02 bc	4.76 ± 0.56 b	5.44 ± 0.39 bc	1.69 ± 0.42 a	ND
18	6.88	*p*-Coumaroyl-glucose	325, 163, 145	5.28 ± 0.07 e	4.51 ± 0.15 d	2.30 ± 0.28 b	1.03 ± 0.05 a	3.59 ± 0.39 c	1.30 ± 0.35 a
19	7.95	*trans*-Fertaric acid	325, 193, 149	7.15 ± 0.48 b	9.92 ± 0.45 d	6.07 ± 0.04 a	8.85 ± 0.23 c	8.48 ± 0.39 c	8.58 ± 0.01 c
20	8.60	*cis*-Fertaric acid	325, 193, 149	1.44 ± 0.08 a	1.51 ± 0.03 a	1.07 ± 0.30 a	1.50 ± 0.06 a	1.30 ± 0.22 a	1.35 ± 0.18 a
21	43.74	Ethyl caffeate	207, 179, 135	1.41 ± 0.05 c	0.68 ± 0.10 ab	0.62 ± 0.35 ab	0.50 ± 0.17 a	1.08 ± 0.04 bc	0.35 ± 0.06 a
		% 2-S-Glutathionyl-caftaric acid		13.73 ± 0.52 a	14.70 ± 0.72 ab	15.92 ± 4.06 ab	18.63 ± 0.57 ab	20.83 ± 0.91 b	47.44 ± 1.65 c
		% Caffeic-type		54.74 ± 1.65 b	51.98 ± 1.07 b	60.02 ± 2.14 c	52.17 ± 0.62 b	60.43 ± 2.08 c	39.19 ± 1.17 a
		% *p*-Coumaric-type		22.94 ± 0.57 d	21.90 ± 0.13 d	16.92 ± 2.27 c	18.85 ± 0.89 c	8.96 ± 0.57 b	3.44 ± 0.66 a
		% Ferulic-type		8.59 ± 0.56 b	11.43 ± 0.48 d	7.14 ± 0.35 a	10.35 ± 0.30 c	9.78 ± 0.61 bc	9.93 ± 0.19 bc

^1^ Peak number used in Figures S1 and S2. ^2^ As quercetin 3-glucoside equivalents. ^3^ As *trans*-caftaric acid equivalents. Abbreviations: ND, not detected. Different letters in the same row indicate that the values are significantly different (ANOVA, Student–Newman–Keuls test, *p* < 0.05).

**Table 2:** Aroma volatile composition (mean value ± standard deviation, *n* = 2) in white wines made from Airén and the novel grape genotypes.

					Airén		Albillo Dorado		Montonera del Casar	
Compounds	m/z	Odor Descriptors	Odorant Series ^1^	Odor Threshold (µg L^−1^)	2015	2016	2015	2016	2015	2016
**C6 Compounds ^2^**					2.24 ± 0.00 bc	2.83 ± 0.59 c	1.12 ± 0.06 a	1.82 ± 0.09 b	0.55 ± 0.05 a	0.52 ± 0.01 a
1-Hexanol ^2^	69	Flower, green, cut grass	II, III	8000 [42]	0.54 ± 0.01 a	0.80 ± 0.18 b	0.39 ± 0.02 a	0.66 ± 0.03 ab	0.42 ± 0.04 a	0.39 ± 0.01 a
*trans*-3-Hexenol ^2^	67	Green	III	400 [46]	0.19 ± 0.00 b	0.17 ± 0.04 b	0.11 ± 0.03 a	0.16 ± 0.00 b	0.07 ± 0.01 a	0.08 ± 0.00 a
*cis*-3-Hexenol ^2^	67	Green, cut grass	III	400 [42]	1.51 ± 0.01 d	1.86 ± 0.37 d	0.62 ± 0.01 b	1.01 ± 0.01 c	0.06 ± 0.00 a	0.05 ± 0.00 a
**Terpenes ^3^**					10.15 ± 2.93 a	10.02 ± 1.78 a	10.19 ± 1.14 a	9.11 ± 1.14 a	10.37 ± 1.16 a	12.41 ± 0.66 a
Linalool ^3^	93	Floral	II	15 [42]	1.80 ± 0.05 a	2.98 ± 0.58 b	1.65 ± 0.22 a	2.78 ± 0.20 b	3.51 ± 0.54 b	3.17 ± 0.19 b
Citronellol ^3^	95	Floral	II	100 [42]	3.20 ± 1.06 a	2.11 ± 0.29 a	4.02 ± 0.78 a	2.21 ± 0.35 a	3.98 ± 1.71 a	2.49 ± 0.04 a
Geraniol ^3^	93	Roses, geranium	II	30 [42]	5.16 ± 1.83 a	4.94 ± 0.91 a	4.53 ± 0.14 a	4.12 ± 0.60 a	2.88 ± 1.08 a	6.75 ± 0.43 a
**Norisoprenoids ^3^**					3.87 ± 0.12 a	3.60 ± 0.54 a	6.75 ± 1.10 b	10.66 ± 3.00 ab	5.56 ± 0.55 ab	7.78 ± 2.67 ab
*β*-Damascenone ^3^	190	Sweet, fruit	I, IV	0.05 [42]	3.84 ± 0.13 a	3.50 ± 0.52 a	6.59 ± 1.11 ab	10.57 ± 2.98 b	5.29 ± 0.33 ab	7.76 ± 2.67 ab
*β*-Ionone ^3^	177	Floral, violet	II	0.09 [45]	0.03 ± 0.01 a	0.10 ± 0.02 a	0.16 ± 0.00 a	0.08 ± 0.01 a	0.27 ± 0.13 a	0.02 ± 0.00 a
**Benzenic Compounds ^2^**					20.58 ± 0.11 a	17.71 ± 5.85 a	24.62 ± 6.67 a	17.87 ± 1.19 a	14.85 ± 1.82 a	11.80 ± 0.29 a
Benzaldehyde ^3^	105	Sweet, cherry, almond	I, IV	2000 [46]	2.62 ± 0.08 b	2.32 ± 0.79 b	1.03 ± 0.17 a	0.98 ± 0.15 a	13.73 ± 0.11 c	44.13 ± 0.31 d
Eugenol ^3^	164	Spices, clove, honey	IV, V, VII	6 [45]	2.14 ± 0.24 b	2.86 ± 0.15 c	0.83 ± 0.31 a	0.93 ± 0.17 a	0.43 ± 0.07 a	0.82 ± 0.18 a
Guaiacol ^3^	124	Medicine, caramel, smoke	IV, VI	10 [45]	9.18 ± 2.40 b	2.14 ± 0.47 a	4.08 ± 0.02 a	2.30 ± 0.66 a	8.71 ± 2.42 b	11.82 ± 1.43 b
Vanillin ^3^	151	Vanilla	V, VII	60 [47]	7.24 ± 1.88 a	7.33 ± 1.26 a	7.26 ± 1.31 a	9.44 ± 2.51 a	8.17 ± 1.34 a	5.75 ± 0.40 a
2-Phenylethanol ^2^	122	Floral, roses	II	10,000 [42]	20.56 ± 0.11 a	17.70 ± 5.85 a	24.60 ± 6.67 a	17.86 ± 1.19 a	14.82 ± 1.81 a	11.74 ± 0.29 a
**Alcohols ^2^**					313.62 ± 14.63 a	284.26 ± 2.73 a	327.07 ± 49.62 a	324.12 ± 34.58 a	307.10 ± 6.56 a	345.45 ± 1.27 a
Methanol ^2^	31	Chemical, medicinal	VI	668,000 [46]	31.05 ± 4.91 a	48.95 ± 1.90 b	44.77 ± 1.11 b	40.53 ± 1.24 ab	76.14 ± 7.74 c	120.37 ± 3.28 d
Isoamyl alcohol ^2^	55	Solvent, fusel	VII	30,000 [42]	228.31 ± 6.31 a	200.17 ± 0.75 a	227.24 ± 43.99 a	231.18 ± 21.49 a	182.94 ± 3.48 a	173.87 ± 0.71 a
3-Ethoxy-propanol ^3^	59	Fruity	I	100 [51]	8.10 ± 0.24 a	3.55 ± 1.63 a	3.58 ± 0.29 a	2.22 ± 1.59 a	24.77 ± 3.38 c	14.58 ± 1.90 b
3-Methylthio-propanol ^2^	106	Cooked, vegetable	VI	500 [42]	0.44 ± 0.02 b	0.25 ± 0.08 ab	0.09 ± 0.03 a	0.22 ± 0.10 a	0.26 ± 0.00 ab	0.25 ± 0.04 ab
Isobutanol ^2^	74	Bitter, green	III, VI	40,000 [42]	53.82 ± 3.37 a	34.88 ± 3.54 a	54.97 ± 6.70 a	52.19 ± 11.76 a	47.74 ± 2.31 a	50.95 ± 1.34 a
**Acids ^2^**					19.04 ± 1.10 a	74.61 ± 8.93 b	14.37 ± 0.23 a	63.56 ± 1.47 b	21.96 ± 0.20 a	75.58 ± 9.13 b
Butyric acid ^2^	73	Rancid, cheese, sweat	VI	173 [45]	0.42 ± 0.03 ab	0.49 ± 0.07 ab	0.08 ± 0.02 a	0.32 ± 0.09 ab	0.70 ± 0.29 b	0.58 ± 0.04 b
Isovaleric acid ^2^	87	Acid, rancid	IV, VI	33 [45]	1.07 ± 0.05 a	1.08 ± 0.30 a	0.45 ± 0.17 a	0.81 ± 0.22 a	0.74 ± 0.06 a	0.91 ± 0.11 a
Hexanoic acid ^2^	60	Sweat	VI	420 [45]	7.23 ± 0.01 ab	12.29 ± 1.83 c	4.99 ± 0.01 a	10.87 ± 0.52 b	7.42 ± 1.34 ab	10.16 ± 0.39 bc
Octanoic acid ^2^	60	Sweat, cheese	VI	500 [45]	7.02 ± 0.87 a	48.48 ± 7.08 b	5.92 ± 0.11 a	38.65 ± 1.28 b	7.72 ± 0.59 a	43.93 ± 4.87 b
Decanoic acid ^2^	73	Rancid fat	VI	1000 [45]	3.31 ± 0.20 a	12.26 ± 0.35 b	2.91 ± 0.19 a	12.91 ± 0.38 b	5.37 ± 0.91 a	19.99 ± 3.71 c
**Aldehydes ^2^**					60.93 ± 9.36 c	76.06 ± 6.43 d	52.11 ± 2.21 bc	41.43 ± 3.46 ab	31.77 ± 6.23 a	32.17 ± 3.58 a
Acetaldehyde ^2^	43	Pungent, ripe apple	I, VI	500 [42]	60.93 ± 9.36 c	76.06 ± 6.43 d	52.11 ± 2.21 bc	41.43 ± 3.46 ab	31.77 ± 6.23 a	32.17 ± 3.58 a
**Esters ^2^**					91.36 ± 0.45 b	58.75 ± 5.45 a	107.65 ± 12.96 c	67.73 ± 5.70 a	111.22 ± 4.60 c	76.24 ± 3.01 ab
Ethyl acetate ^2^	70	Fruity, solvent	I, VI	7500 [42]	62.43 ± 1.94 ab	43.35 ± 5.13 a	69.37 ± 14.74 ab	50.21 ± 5.49 ab	73.13 ± 5.40 b	60.28 ± 2.18 ab
Isoamyl acetate ^2^	61	Banana	I	30 [45]	17.31 ± 0.56 c	7.08 ± 1.13 a	11.89 ± 0.81 b	6.97 ± 0.60 a	16.19 ± 1.79 c	6.32 ± 0.41 a
2-Phenylethyl acetate ^2^	104	Floral, roses	II	250 [42]	0.25 ± 0.00 a	0.26 ± 0.03 a	0.25 ± 0.01 a	0.26 ± 0.02 a	0.20 ± 0.01 a	0.26 ± 0.02 a
Ethyl lactate ^2^	75	Acid, medicine	VI	154,636 [47]	8.84 ± 2.06 a	5.06 ± 0.52 a	23.75 ± 2.88 b	7.41 ± 0.72 a	19.24 ± 2.67 b	6.47 ± 0.44 a
Ethyl butyrate ^2^	88	Fruity	I	20 [42]	0.62 ± 0.01 b	0.25 ± 0.06 a	0.69 ± 0.11 b	0.30 ± 0.04 a	0.86 ± 0.06 c	0.26 ± 0.01 a
Ethyl hexanoate ^2^	88	Green apple	I	14 [45]	0.70 ± 0.00 a	1.03 ± 0.12 b	0.57 ± 0.04 a	0.89 ± 0.06 b	0.53 ± 0.00 a	0.85 ± 0.00 b
Ethyl octanoate ^2^	88	Sweet, fruity	I, II, IV	5 [45]	1.06 ± 0.01 abc	1.47 ± 0.19 c	0.98 ± 0.13 ab	1.34 ± 0.17 bc	0.92 ± 0.00 a	1.39 ± 0.04 c
Ethyl decanoate ^2^	88	Sweet, fruity	I, IV	200 [45]	0.17 ± 0.00 a	0.27 ± 0.01 b	0.15 ± 0.01 a	0.36 ± 0.07 c	0.18 ± 0.02 a	0.41 ± 0.01 c
**Furanic Compounds ^3^**					13.22 ± 0.07 ab	27.34 ± 4.71 b	3.24 ± 2.12 a	15.24 ± 8.36 ab	7.50 ± 1.95 a	17.40 ± 3.93 ab
Furaneol ^3^	128	Burnt sugar, caramel	IV	5 [52]	13.22 ± 0.07 ab	27.34 ± 4.71 b	3.24 ± 2.12 a	15.24 ± 8.36 ab	7.50 ± 1.95 a	17.40 ± 3.93 ab
**Lactones ^3^**					58.92 ± 4.11 a	149.15 ± 37.65 b	51.00 ± 1.98 a	144.67 ± 39.34 b	75.53 ± 12.25 a	175.37 ± 3.37 b
γ-Butyrolactone ^3^	86	Sweet, toast, caramel	IV	35,000 [53]	42.76 ± 3.35 a	37.74 ± 8.96 a	39.82 ± 3.60 a	66.94 ± 20.94 a	57.28 ± 2.69 a	106.08 ± 3.57 b
γ-Nonalactone ^3^	85	Coconut	IV	30 [45]	1.52 ± 0.14 a	3.29 ± 0.68 b	2.68 ± 0.25 b	7.19 ± 0.50 d	4.88 ± 0.58 c	6.29 ± 0.13 d
δ-Dodecalactone ^3^	114	Coconut	IV	88 [46]	14.64 ± 0.90 a	108.12 ± 28.01 c	8.50 ± 1.87 a	70.54 ± 17.90 b	13.37 ± 8.98 a	63.00 ± 0.07 b

^1^ I = fruity; II = floral; III = green, fresh; IV = sweet; V = spicy; VI = fatty; VII = others. ^2^ mg L^−1^. ^3^ µg L^−1^. Different letters in the same row indicate that the values are significantly different (ANOVA, Student–Newman–Keuls test, *p* < 0.05).

## Supplementary Materials

The following are available online at https://www.mdpi.com/2304-8158/9/9/1282/s1, Figure S1: Flavonol profiles (HPLC DAD-chromatograms at 360 nm) in white wines made from Airén and novel grape genotypes: (a) Airén, (b) Albillo Dorado, (c) Montonera del Casar. Peak numbering is as in Table 1, Figure S2: Hydroxycinnamic acid derivative profiles (HPLC DAD-chromatograms at 320 nm) in white wines made from Airén and novel grape genotypes: (a) Airén, (b) Albillo Dorado, (c) Montonera del Casar. Peak numbering is as in Table 1, Table S1: Calibration data of commercial standards used for the quantification of flavonols and hydroxycinnamic acid derivatives in white wines made from Airén and novel grape genotypes, Table S2: Physicochemical parameters, global phenolic composition and chromatic characteristics (mean value ± standard deviation, *n* = 2) in white wines made from Airén and novel grape genotypes, Table S3: Flavan-3-ol monomer, dimer and stilbene molar percentage and total concentration (mean value ± standard deviation, *n* = 2) in white wines made from Airén and novel grape genotypes.
