# Supplementary material for: Comprehensive Chemical and Sensory Assessment of Wines Made from White Grapes of Vitis vinifera Cultivars Albillo Dorado and Montonera del Casar: A Comparative Study with Airén
Source: Foods. 2020 Sep 12;9(9):1282. doi: 10.3390/foods9091282 (PMC7556037; doi:10.3390/foods9091282)
Supplement: Supplementary file 1 [file foods-09-01282-s001.pdf]

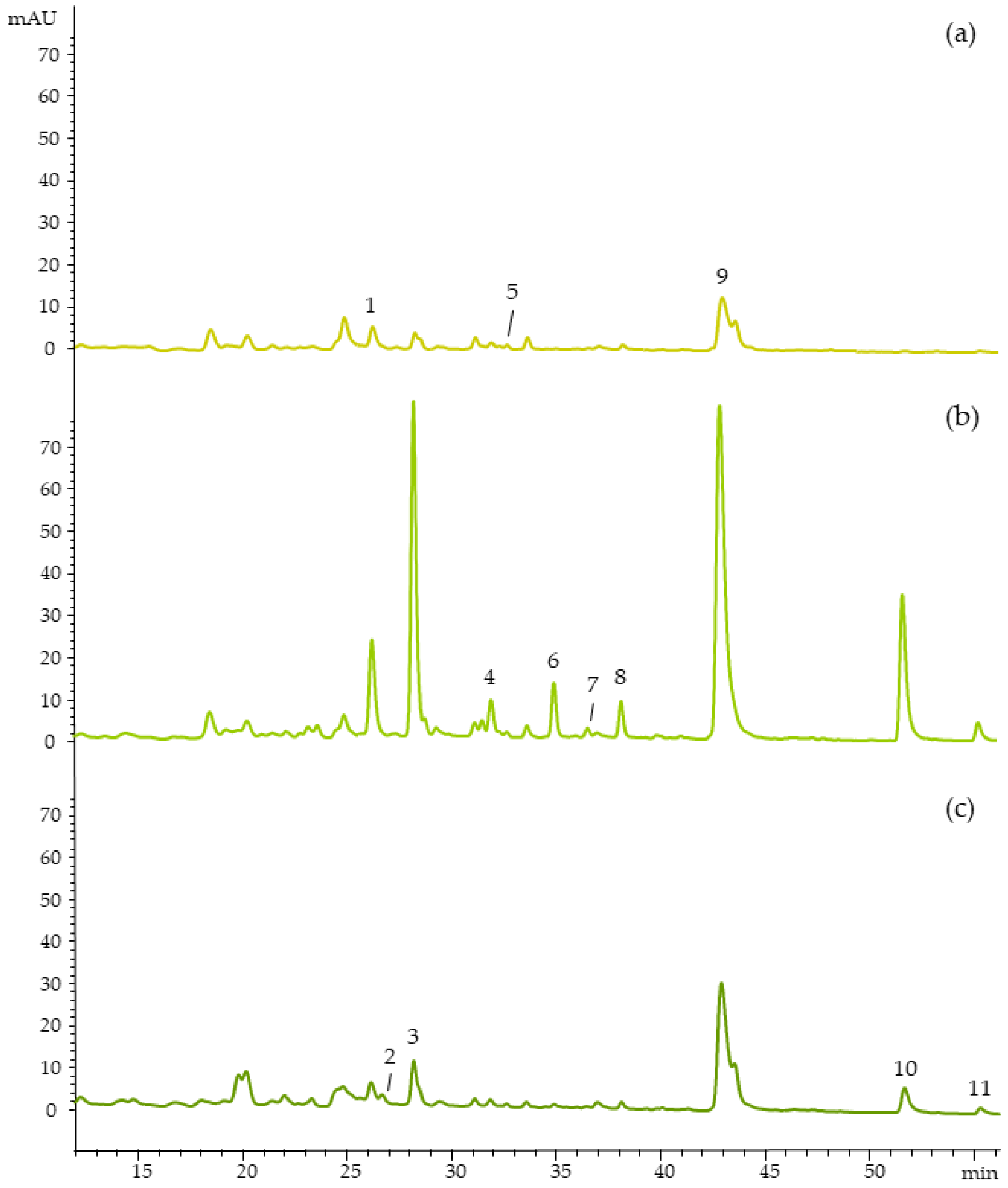


**Figure S1.** Flavonol profiles (HPLC DAD-chromatograms at 360 nm) in white wines made from Airén and novel grape genotypes: (**a**) Airén, (**b**) Albillo Dorado, (**c**) Montonera del Casar. Peak numbering is as in Table 1.

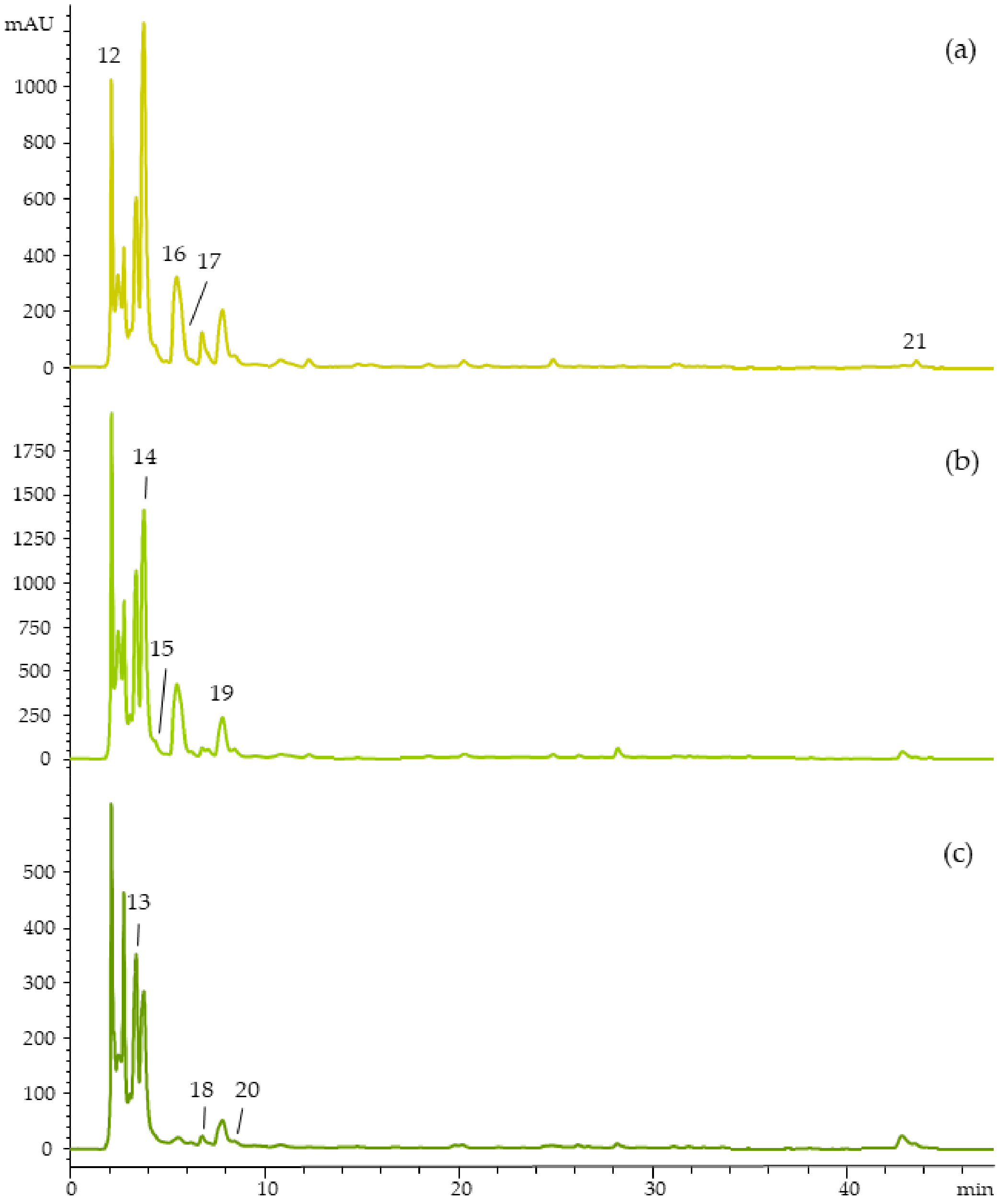


**Figure S2.** Hydroxycinnamic acid derivative profiles (HPLC DAD-chromatograms at 320 nm) in white wines made from Airén and novel grape genotypes: (**a**) Airén, (**b**) Albillo Dorado, (**c**) Montonera del Casar. Peak numbering is as in Table 1.

**Table S1.** Calibration data of commercial standards used for the quantification of flavonols and hydroxycinnamic acid derivatives in white wines made from Airén and novel grape genotypes.

| Compound | Linear range (mg/L) | Linear curve | $R^2$ |
|---|---|---|---|
| Quercetin 3-glucoside | 10 - 100 | y = 0.0053 + 0.0009 | 0.9998 |
| *trans*-Caftaric acid | 5 - 100 | y = 0.0033 – 0.2715 | 0.9996 |

**Table S2.** Physicochemical parameters, global phenolic composition and chromatic characteristics (mean value ± standard deviation, n=2) in white wines made from Airén and novel grape genotypes.

| | **Airén** | | **Albillo Dorado** | | **Montonera del Casar** | |
|---|---|---|---|---|---|---|
| | **2015** | **2016** | **2015** | **2016** | **2015** | **2016** |
| **Physicochemical parameters** | | | | | | |
| Relative density | 0.987 ± 0.002 a | 0.989 ± 0.000 b | 0.987 ± 0.001 a | 0.989 ± 0.000 b | 0.991 ± 0.000 b | 0.989 ± 0.000 b |
| Alcoholic strength (%, v/v) | 12.35 ± 0.03 ab | 11.82 ± 0.01 a | 13.76 ± 1.08 b | 12.77 ± 0.05 ab | 11.25 ± 0.06 a | 13.37 ± 0.03 b |
| Total acidity ($g\ L^{-1}$) | 3.74 ± 0.01 a | 3.97 ± 0.20 a | 3.79 ± 0.03 a | 4.58 ± 0.13 b | 4.79 ± 0.07 b | 5.58 ± 0.01 c |
| pH | 3.62 ± 0.02 a | 3.49 ± 0.01 a | 3.54 ± 0.08 a | 3.55 ± 0.01 a | 3.52 ± 0.01 a | 3.61 ± 0.02 a |
| Volatile acidity ($g\ L^{-1}$) | 0.23 ± 0.03 a | 0.28 ± 0.03 a | 0.43 ± 0.01 b | 0.28 ± 0.00 a | 0.22 ± 0.01 a | 0.44 ± 0.00 b |
| Total $SO_2$ ($mg\ L^{-1}$) | 141.00 ± 5.66 c | 169.00 ± 1.41 d | 130.00 ± 2.83 bc | 104.00 ± 2.83 a | 120.00 ± 11.31 b | 119.00 ± 1.41 b |
| Glucose + Fructose ($g\ L^{-1}$) | 0.24 ± 0.01 c | 0.22 ± 0.01 ab | 0.20 ± 0.00 ab | 0.18 ± 0.00 a | 0.30 ± 0.04 c | 0.35 ± 0.01 d |
| Malic acid ($g\ L^{-1}$) | 1.17 ± 0.03 a | 1.07 ± 0.01 a | 1.02 ± 0.28 a | 1.39 ± 0.13 ab | 1.69 ± 0.05 b | 1.65 ± 0.07 b |
| Tartaric acid ($g\ L^{-1}$) | 1.26 ± 0.03 a | 1.23 ± 0.08 a | 1.60 ± 0.13 a | 1.73 ± 0.19 a | 2.18 ± 0.28 b | 2.27 ± 0.00 b |
| Succinic acid ($g\ L^{-1}$) | 0.54 ± 0.04 ab | 0.40 ± 0.03 a | 0.66 ± 0.12 b | 0.57 ± 0.03 ab | 0.59 ± 0.05 ab | 0.44 ± 0.00 ab |
| Citric acid ($g\ L^{-1}$) | 0.21 ± 0.01 c | 0.17 ± 0.00 b | 0.17 ± 0.01 b | 0.11 ± 0.00 a | 0.43 ± 0.00 e | 0.37 ± 0.01 d |
| Glycerin ($g\ L^{-1}$) | 5.38 ± 0.07 ab | 5.11 ± 0.03 a | 6.63 ± 0.23 e | 5.75 ± 0.14 c | 5.60 ± 0.05 bc | 6.34 ± 0.01 d |
| **Polyphenols and color** | | | | | | |
| Total polyphenols ($g\ L^{-1}$)[1] | 0.41 ± 0.06 a | 0.35 ± 0.02 a | 0.40 ± 0.05 a | 0.34 ± 0.02 a | 0.35 ± 0.03 a | 0.30 ± 0.02 a |
| Condensed tannins ($mg\ L^{-1}$)[2] | 75.14 ± 19.85 c | 74.41 ± 13.34 c | 54.65 ± 13.19 b | 58.69 ± 10.37 b | 16.24 ± 2.22 a | 10.48 ± 2.96 a |
| Lightness (L*) | 99.32 ± 0.00 a | 99.15 ± 0.07 a | 98.70 ± 0.26 a | 98.65 ± 0.00 a | 98.83 ± 0.42 a | 98.22 ± 0.07 a |
| Red-greenness (a*) | -0.83 ± 0.05 c | -0.75 ± 0.04 c | -0.99 ± 0.02 bc | -1.03 ± 0.01 bc | -1.10 ± 0.04 b | -1.22 ± 0.11 a |
| Yellow-blueness (b*) | 4.27 ± 0.32 a | 3.96 ± 0.08 a | 5.43 ± 0.44 b | 6.33 ± 0.24 bc | 6.23 ± 0.55 bc | 7.05 ± 0.36 c |
| Chroma (C*) | 4.35 ± 0.30 a | 4.03 ± 0.07 a | 5.52 ± 0.43 b | 6.42 ± 0.23 bc | 6.33 ± 0.54 bc | 7.16 ± 0.38 c |
| Hue angle (h*) | 101.02 ± 1.41 a | 100.77 ± 0.69 a | 100.35 ± 0.76 a | 99.29 ± 0.45 a | 100.05 ± 1.20 a | 99.79 ± 0.31 a |

[1] As gallic acid equivalents. [2] As (-)-epicatechin equivalents. Different letters in the same row indicates that the values are significantly different (ANOVA, Student–Newman–Keuls test, $p<0.05$).

**Table S3.** Flavan-3-ol monomer, dimer and stilbene molar percentage and total concentration (mean value ± standard deviation, n=2) in white wines made from Airén and novel grape genotypes.

| Compounds | m/z pairs[4] | Airén 2015 | Airén 2016 | Albillo Dorado 2015 | Albillo Dorado 2016 | Montonera del Casar 2015 | Montonera del Casar 2016 |
|---|---|---|---|---|---|---|---|
| **Flavan-3-ol monomers** (mg $L^{-1}$)[1] | | 16.47 ± 1.41 c | 15.80 ± 0.59 c | 7.91 ± 1.95 b | 14.05 ± 0.21 c | 1.81 ± 0.26 a | 1.51 ± 0.14 a |
| (+)-Catechin | 289/137; 289/164 | 56.18 ± 1.07 a | 56.60 ± 0.04 a | 55.23 ± 1.34 a | 53.77 ± 1.07 a | 68.01 ± 1.98 b | 70.42 ± 3.18 b |
| (-)-Epicatechin | 289/137; 289/164 | 21.50 ± 0.87 f | 14.13 ± 0.12 c | 16.55 ± 0.44 d | 18.91 ± 0.51 e | 11.67 ± 1.28 b | 7.27 ± 0.90 a |
| (-)-Gallocatechin | 305/109; 305/137 | 15.77 ± 0.68 b | 21.92 ± 0.20 c | 17.46 ± 1.59 b | 17.76 ± 0.01 b | 5.60 ± 1.12 a | 4.57 ± 0.14 a |
| (-)-Epigallocatechin | 305/109; 305/137 | 1.40 ± 0.11 a | 1.81 ± 0.05 b | 1.80 ± 0.16 b | 2.06 ± 0.16 b | ND | ND |
| (-)-Epicatechin gallate | 441/245; 441/289 | 0.04 ± 0.02 a | 0.06 ± 0.00 a | 0.28 ± 0.07 ab | 0.19 ± 0.03 a | 0.55 ± 0.26 b | ND |
| Monoglucosides | 451/289; 451/245 | 5.12 ± 0.61 a | 5.48 ± 0.07 a | 8.68 ± 0.41 a | 7.32 ± 0.38 a | 14.17 ± 2.09 b | 17.74 ± 2.15 c |
| **Flavan-3-ol dimers** (mg $L^{-1}$)[2] | | 13.00 ± 3.17 b | 10.12 ± 1.87 b | 2.17 ± 0.12 a | 4.93 ± 0.92 a | 0.19 ± 0.01 a | 0.12 ± 0.01 a |
| Procyanidin B1 | 577/425; 577/407 | 36.05 ± 2.90 a | 60.26 ± 1.28 b | 60.98 ± 1.30 b | 64.03 ± 1.18 b | 62.63 ± 1.20 b | 78.33 ± 0.39 c |
| Procyanidin B2 | 577/425; 577/407 | 7.28 ± 0.92 a | 7.58 ± 0.26 a | 15.25 ± 0.55 a | 14.61 ± 0.35 a | 17.06 ± 6.14 a | 11.57 ± 2.38 a |
| Procyanidin B4 | 577/425; 577/407 | 30.51 ± 4.10 c | 11.63 ± 0.65 b | 2.71 ± 1.01 a | 2.57 ± 0.04 a | ND | ND |
| Other dimers | 577/425; 577/407 | 26.00 ± 0.01 b | 20.14 ± 0.39 ab | 20.04 ± 1.07 ab | 17.65 ± 1.05 ab | 21.31 ± 7.34 ab | 10.10 ± 1.99 a |
| Galloylated dimers | 729/577; 729/289 | 0.16 ± 0.06 a | 0.39 ± 0.03 a | 1.02 ± 0.24 b | 1.14 ± 0.26 b | ND | ND |
| **Stilbenes** (mg $L^{-1}$)[3] | | 0.19 ± 0.00 b | 0.13 ± 0.02 ab | 0.13 ± 0.05 ab | 0.32 ± 0.02 c | 0.09 ± 0.01 a | 0.07 ± 0.00 a |
| *cis*-Resveratrol | 227/143; 227/185 | 61.79 ± 0.00 d | 45.06 ± 3.47 b | 38.66 ± 2.35 ab | 38.55 ± 1.83 ab | 32.01 ± 0.48 a | 51.17 ± 2.78 c |
| *cis*-Piceid | 389/227; 227/185 | 28.58 ± 0.21 a | 36.55 ± 3.52 b | 35.82 ± 0.22 b | 42.94 ± 0.69 c | 44.86 ± 3.23 c | 33.52 ± 1.15 ab |
| *trans*-Piceid | 389/227; 227/185 | 9.63 ± 0.22 a | 18.40 ± 0.05 bc | 25.51 ± 2.13 d | 18.51 ± 1.15 bc | 23.13 ± 2.75 cd | 15.31 ± 1.63 b |

[1] As (+)-catechin equivalents. [2] As procyanidin B1 equivalents. [3] As *trans*-resveratrol equivalents. [4] Mass transition pairs data for MRM scan used in the identification of the compounds. Abbreviations: ND, not detected. Different letters in the same row indicates that the values are significantly different (ANOVA, Student–Newman–Keuls test, $p<0.05$).
